# Characteristics of the King-Devick test in the assessment of concussed patients in the subacute and later stages after injury

**DOI:** 10.1371/journal.pone.0183092

**Published:** 2017-08-31

**Authors:** Arsenije Subotic, Windsor Kwan-Chun Ting, Michael D. Cusimano

**Affiliations:** 1 Division of Neurosurgery, Department of Surgery, Injury Prevention Research Office, St. Michael’s Hospital, Toronto, Ontario, Canada; 2 University of Toronto, Toronto, Ontario, Canada; Cleveland Clinic, UNITED STATES

## Abstract

Although the King-Devick (K-D) test has been used frequently in assessing sports related concussion early after injury, its characteristics over time after injury and in patients with prolonged persistent symptoms are unknown. The purpose of this paper was to: evaluate the ability of the K-D Test to distinguish patients seen early after concussion from those with symptoms persisting more than 3 months compared to controls, assess changes in the K-D test times over time after concussion, and determine the relationship of K-D times to the Stroop Color and Word Test scores. We performed cross-sectional comparisons of patients with recent concussive brain injury (acute group) and those with symptoms persisting more than 3 months to healthy controls on the K-D test, the Sports Concussion Assessment Tool 3 (SCAT3), and the Stroop Color and Word Test. Longitudinal comparisons of the acute group over time within the first month after injury were also made. Post-concussive syndrome (PCS) patients had significantly higher K-D times compared to controls (p = 0.01), while the acute group did not differ from controls(p = 0.33). K-D times at the second visit for the acute group were similar to those of controls (54.7 vs. 49.6, p = 0.31). While SCAT3 scores improved over time in the acute group, the K-D scores did not change between the first and second visit (55.2 vs. 54.7, p = 0.94). K-D scores correlated significantly with the Stroop scores for all three participant groups. The K-D test is likely useful very early after concussion in conjunction with baseline scores, and while scores in PCS patients remain elevated, they can be confounded by factors such as pre-morbid depression and medication use. High correlations with Stroop scores also suggest that performance on the K-D test can by proxy provide additional insight about cognitive function and predict performance on more cognitively demanding tasks.

## Introduction

Rising public concern regarding the occurrence of mild traumatic brain injuries (mTBIs) including concussion in sports has led to the development of tools that can help assist in the screening, diagnosis and follow-up of these injuries. The Sport Concussion Assessment Tool 3 (SCAT3), is frequently administered to concussed athletes to assess the number and degree of severity of symptoms pertaining to mTBI [1]. Symptoms on the SCAT3 can be counted and given a rating of severity, but these scores share the same limitations of all self-reported scales: they may be difficult to assess in the presence of preexisting symptoms and are open to potential bias as athletes may under or over-report their symptoms.

Due to such limitations, other tests have been investigated that can help aid clinicians in the screening and diagnosis of those suspected of brain injury. There is increasing interest in the King-Devick test (K-D) as a screening tool for concussion and mTBI. The K-D test requires that participants read a series of three test cards of numbers, which become progressively more difficult to read, as quickly as they can [2]. The total time to complete all three test cards, and the number of errors committed are included in the total score. Studies have shown the K-D test to be a sensitive marker of brain injury by detecting attentional deficits and impaired saccadic eye movements, which have been associated with higher (worse) K-D scores among athletes [3–6]. The K-D test has become a popular sideline screening tool for concussions in sports, since it is easy to administer and usually takes less than two minutes to complete. Several studies have used it in hockey league cohorts [7–9], boxing and mixed martial arts (MMA) competitions [10,11], football games [6, 12–15], as well as rugby league competitions [5, 15–18]. Despite its extensive use immediately following a suspected TBI, the efficacy of the K-D test in tracking symptom resolution longitudinally has been less widely investigated. Tjarks et al. [19] conducted a longitudinal study on concussed patients presenting at a sports clinic, but did not focus on patients affected by non-sports related injuries or those experiencing post-concussive syndrome (PCS) for more than 3 months. Most of the first visits after concussion occurred in the 6–10 day interval (40%) and after 30 days (26%), with 6% of the patients coming in the 1–5 day interval [19]. They found that K-D scores improved at each visit over the four-visit study period [19]. In addition, only Silverberg [20] and Benedict et al. [21] have examined the validity of the K-D test in assessing non-sports related TBI, but neither examined whether the K-D test could be used as an indicator of patient recovery over time after the injury. To our knowledge, previous studies have also not explored the option of correlating performance on the K-D test with tests assessing executive functions such as inhibitory control to see if the K-D test can by-proxy provide more understanding into cognitive function after head injuries.

The purpose of this study was three-fold. First, we wanted to determine if the K-D test could accurately distinguish between non-injured healthy controls, acutely injured patients, and those with persistent post-concussive syndrome with symptoms lasting more than 3 months (PCS). We hypothesized that higher (worse) K-D test scores would be seen in the acutely injured group and PCS patients compared to healthy controls. Second, we aimed to determine if K-D test scores changed over time from the original injury among the acute group patients and if it correlated to symptom resolution over time. We hypothesized that K-D scores would correlate with self-report symptom scores and show improvement over time as symptoms resolved. Our third aim was to correlate and compare K-D scores with assessments of selective attention and processing speed, namely the Stroop Color and Word Test. Our hypothesis was that scores of the Stroop assessment would negatively correlate with K-D scores.

## Methods

The study was approved by the Research Ethics Board (REB) at St. Michael’s Hospital (SMH). Patients were recruited from the Emergency Department, Head Injury Clinic, and the inpatient Neurosurgery Ward. Healthy controls were recruited through word of mouth to family members or relatives of participants, as well as staff at SMH in the Emergency Department. Capacity for consent was determined by following the PATIENT Modified Aid to Capacity Evaluation (ACE) Screening Tool utilized by SMH. Participants must have been able to: communicate, understand their current medical condition, understand the purpose of the research study, understand the option of declining to participate (with no impact on medical care), understand the risks of participating, and make a decision that is not substantially based upon hallucinations, delusions, or cognitive signs of depression. If any of these criteria were not met, we did not proceed to consent. After this, written informed consent was obtained, after which participants were assessed for eligibility according to the inclusion/exclusion criteria, outlined below.

### Inclusion/Exclusion criteria

Acute group patients were defined as patients who had suffered a non-penetrating head injury and exhibited a GCS score of 13–15 at the time of recruitment. PCS patients were defined as those who had sustained a mTBI three or more months prior to their first testing visit, and were still experiencing ongoing symptoms. Inclusion criteria included: being the age of 16 or over, being able to provide informed written consent, and having sufficient fluency in English. Healthy controls were matched to mTBI patients according to age (±2.5years), sex, and years of education (±2.5 years). Controls participants must also not have had a history of prior head injuries. All participants were also asked to complete a screening form to ensure their eligibility. Participants were excluded if they had the following medical conditions: history of multiple sclerosis, prior hydrocephalus, prior brain irradiation, prior stroke, comorbid early dementia, comorbid Parkinson’s disease, comorbid uncontrolled diabetes, comorbid eye disease causing strabismus, comorbid non-affective psychiatric illness, active substance abuse requiring treatment, comorbid alcohol related dementia, and comorbid immune-compromised (HIV/AIDS or taking immunosuppressive therapy). In addition, participants were excluded if they were unable to provide consent because of being medically unstable or intoxicated at the time of recruitment. At the time of screening we also asked for current medication use.

### Participant assessments

PCS participants and controls were asked to come in for one visit, and those in the acute injury group were asked to come in for two visits. PCS and healthy controls were asked to come in at their earliest possible convenience. The first visit for acute participants was conducted within 10 days of the injury, while the second was conducted at 2–4 weeks post-injury (PI). The minimum time between the first and second visit was seven days. At each visit, participants completed the K-D test, the SCAT3 Symptom Evaluation, and the Stroop Color and Word Test. The descriptions of these tests are outlined below.

### King-Devick (K-D) test

The K-D test is a saccadic eye measurement test that relies on the principle of rapid number naming [1–15]. The test contains a demonstration (practice) card and three test cards of variably spaced single-digit numbers [1–3, 5]. Participants are asked to read out aloud the numbers from left to right as quick as they can without making any errors [1–15]. The time taken for each card as well as the number of errors was recorded and summed to provide the total K-D score [1–15]. The test usually takes less than two minutes to complete [3,5].

### SCAT3 symptom evaluation

The symptom evaluation is composed of 22 different symptoms, each of which are rated on a scale of 0–6, with 0 indicating absence of symptoms and 6 being most severe [17, 21]. The total score is out of 22, with symptoms being counted towards the total score if they are non-zero values [17, 21]. The symptom severity score is obtained by summing the values of the individual symptom scores, resulting in a maximum score of 132[17, 21].

### Stroop Color and word test

The Stroop Color and Word Test is a neuropsychological test used to measure executive control and selective attention [22–25]. Participants are asked to name the colour of the words presented as fast as they can [22–25]. This becomes more difficult to do if the colour is incongruent with the word [23–25]. It is easier to name the word ‘red’ if it is printed in red than if it is green. In the latter situation, this conflict slows responding which leads to the ‘Stroop effect’ [23–25]. The scores on the task reflect how well participants can selectively direct attention to task relevant features while ignoring task-irrelevant features [23–25]. In our study, participants were asked to read the colour of words (blue, red, green) on a sheet of paper as fast as they can. There were 10 rows, each containing 10 words, resulting in a total of 100 words. The time limit was 45 seconds. If participants finished early, they were asked to read again from the beginning. The total number of words and number of errors are included in the final score.

### Statistical analyses

Descriptive statistics were used to describe the participants. Differences in K-D Test, SCAT3, and the Stroop Color and Word Test scores between groups and time intervals were compared using one-way ANOVA. Post-hoc analysis using Fisher’s Least Significant Difference was performed after one-way ANOVA. Paired t-tests were used to do compare scores between the first and second visit for the acute group. Pearson correlation coefficients were used to calculate the correlation between the test scores. All analysis was conducted using Stata 13. Statistical significance was set at α = 0.05.

## Results

### Participant characteristics

In total, 17 acute, 28 PCS and 18 controls participants completed the K-D test. The demographic data pertaining to each participant group are outlined in Table 1.

**Table 1:** Basic demographic characteristics of acute mTBI, PCS, and control participant groups.

		Acute mtBI	PCS	Controls
**N**				
	First Visit	17	28	18
	Second Visit	17	-	-
**Age**	Mean ± SD	42.7 ±14.47	46.1 ±12.8	36.9 ±16.6
**Sex, (%)**				
	Male	47%	18%	44%
	Female	53%	82%	56%
**Education**	Mean ± SD	14.9 ± 3.2	16.4 ± 3.2	15.4 ± 1.8
**Mechanism of injury (%)**				
	Fall	47	36	-
	Sports	12	4	-
	Unintentional contact with an object	29	18	-
	MVC	6	38	-
	Intentional Assault	6	4	-
**Medication use, N (% of total population)**				
	Benzodiazepines	0 (0)	3 (11)	0 (0)
	Antidepressants	2 (12)	6 (21)	2 (11)
	Headache Relief	1 (6)	5 (18)	1 (6)
**Initial Glasgow Coma Scale (GCS) in ED, N**				
	13	1	0	0
	14	0	0	0
	15	16	28	18
**History of Concussion, (%)**		47	50	-

Abbreviations: SD, standard deviation; MVC, motor vehicle collision; ED, emergency department

Acute participants were assessed at a median of 5 days (Interquartile Range, IQR = 3–7) PI for their first visit. The second visit occurred at a median of 24 (IQR = 16–30) days PI. The single visit for the PCS patients occurred at a median of 366 days (IQR = 164–659) after injury. It was reported that 13/28 (44%) of PCS participants were using medication to relieve headache, anxiety and/or depression due to their injury, compared to 3/17 (18%) of acute participants (Table 1). Of these 12 participants in the PCS group, 3 were using benzodiazepines, 6 were using antidepressants, and 5 were using medications (ibuprofen, acetyl salicylic acid, acetaminophen) to relieve headaches.

### K-D and SCAT3 score characteristics

For the first visit, K-D mean scores were 55.2 ± 12.5s for acute group, 64.1 ± 23.4s for PCS, and 49.6 ± 11.2s for healthy controls (Table 2). PCS participants took significantly longer than controls(p = 0.01), but were not significantly different from the acute group (p = 0.13). K-D scores were not different from the first to second visit in the acute group (55.2 ± 12.5 s versus 54.7 ± 15.1s, p = 0.94), and nor were they different from controls (p = 0.31). The total symptom score and symptom severity scores were higher for the acute group and PCS group compared to controls on the first visit (p<0.0001). For the acute group, symptom scores and severity scores improved significantly from the first visit to the second (13.2 ± 6.4 vs. 7.5 ± 5.9, p = 0.01) (32.6 ± 19.2 vs. 13.4 ± 12.3, p = 0.002) (Table 2).

**Table 2:** Summary of test scores by group and by visit.

		Acute mTBI	PCS	Controls
**K-D Times, Mean ± SD (s)**				
	First Visit	55.2 ± 12.5	64.1 ± 23.4*	49.6 ± 11.2
	Second Visit	54.7 ± 15.1	-	-
**Symptom Total Score, Mean ± SD**				
	First Visit	13.2±6.4	15.2 ± 6.06	1.4 ± 2.5
	Second Visit	7.5 ± 5.9	-	-
**Symptom Severity Score, Mean ± SD**				
	First Visit	32.6 ±19.2	44.4 ± 31.3	2.3 ± 4.2
	Second Visit	13.4 ±12.3	-	-
**Stroop Color and Word Test, Mean number of words ± SD**				
	First Visit	69.4 ± 15.9	72.3 ± 11.6	85.1 ± 12.4
	Second Visit	70.5 ± 14.1	-	-

*Post-hoc analysis. F-value = 4.66, P = 0.01.

**Fig 1:**
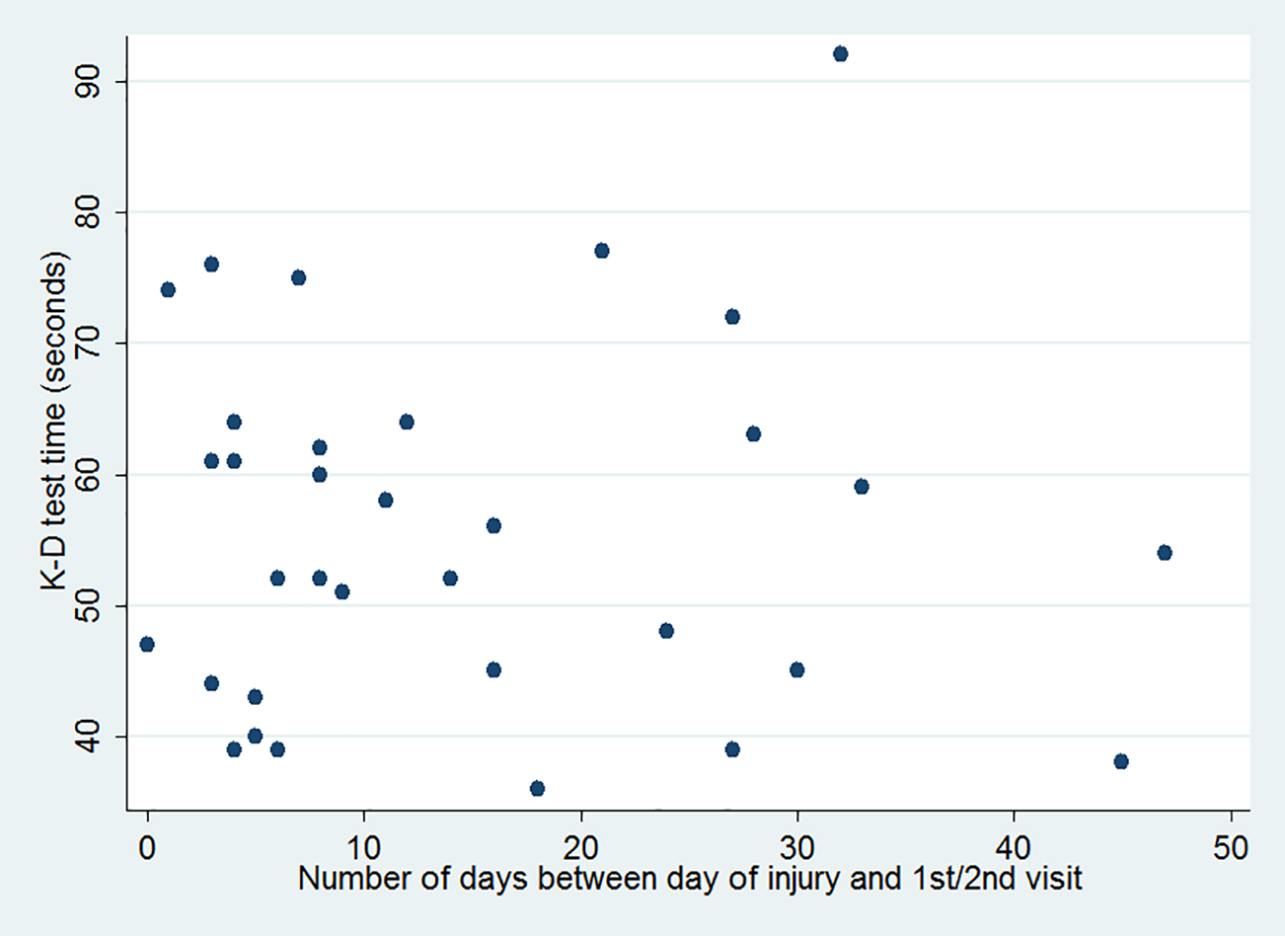
Relationship between K-D scores and number of days between injury and test visit in the acute group.

**Fig 2:**
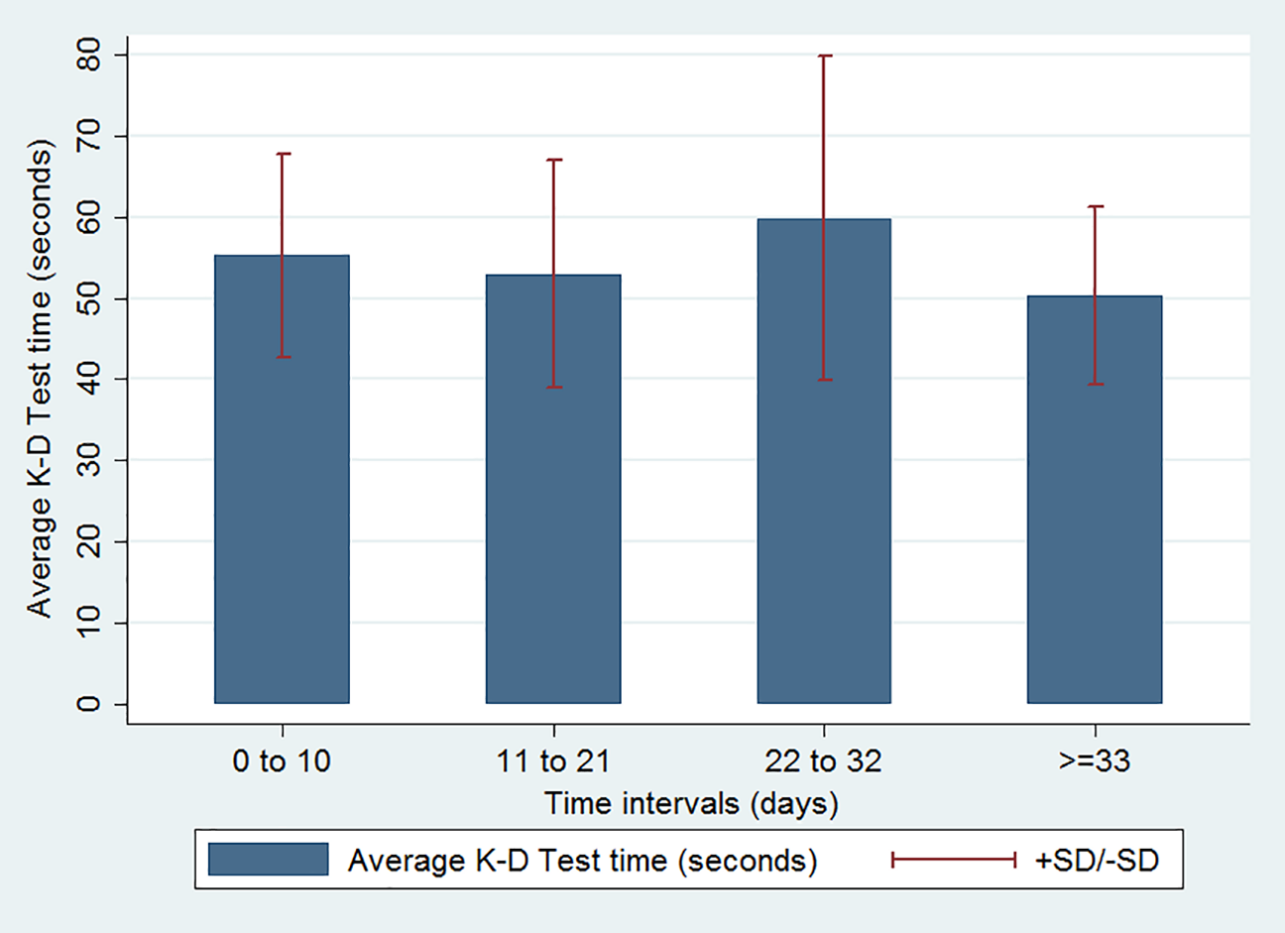
Average K-D score for each time interval after injury in the acute group. Abbreviations: SD, standard deviation. Symbols: > =, greater than or equal to.

We also performed an analysis of K-D values over time to show the pattern of scores in the acute group. A scatterplot (Fig 1) showing K-D scores and the time between date of injury and test visits showed no significant correlation or relationship. In addition, we stratified the time between the date of injury and the first/second visit into four time intervals (Fig 2) to more precisely characterize K-D performance as a function of time. Again, there were no significant differences between each time interval in terms of K-D scores.

### K-D and Stroop Color and word test characteristics

For the Stroop Color and Word Test, the acute group at the first visit got fewer words correct than the controls (69.4 ± 15.9 versus 85.1 ± 12.4, p<0.0001, Table 2). Similarly, at the second visit, the acute group differed significantly from controls (p = 0.003), but did not exhibit an improvement from the first visit (p = 0.82). The PCS group also scored significantly lower than controls (p = 0.002). A strong, inverse correlation was found for all three groups (Table 3).

**Table 3:** Correlations of K-D test scores with other assessment scores.

		Symptom Total Score	Symptom Severity Score	Stroop Color and Word Test
Acute: 1st Visit				
	r	-0.53	-0.43	-0.89
	p	0.03	0.08	<0.0001
Acute: 2nd Visit				
	r	0.05	0.15	-0.78
	p	0.86	0.58	0.0002
PCS				
	r	0.06	0.07	-0.75
	p	0.76	0.71	<0.0001
Healthy Control				
	r	0.30	0.22	-0.60
	p	0.23	0.39	0.001

## Discussion

### Major findings

We found that PCS patients performed significantly worse than controls and moderately worse than our acute group of patients on the K-D test. A number of potential explanations exist for this. Our PCS patients sustained their injuries more frequently in motor vehicle crashes (MVC) than the acute group and since MVC injuries are higher velocity events than those in sports, they are likely also linked with increased concussion severity and the prolongation of symptom burden [26–31]. Those suffering from PCS may have experienced greater damage to cortical and subcortical structures in the prefrontal cortex, thus resulting in ongoing cerebral impairment and slowing of saccades beyond the usual 1-3-month period in which most decrements in cognitive function resolve [26, 32]. In addition, the PCS group was more medicated for conditions like depression or anxiety which can contribute to prolongation of K-D times and post-concussive symptoms. [30, 32]. Reilly [33] found that the usage of benzodiazepines and other sedatives used to relieve anxiety have been associated with a decrease in saccadic acceleration and velocity and an increase in saccadic latency, or the time interval between two consecutive saccades, which would slow down saccadic eye movements [33]. However, it was also reported that the usage of antidepressants had no significant effect on saccadic eye movements [33]. So, it is difficult to be certain whether tests of saccadic eye movements can be used as sensitive and objective markers of brain dysfunction following injury, particularly in patients on confounding medications for depression or anxiety [33]. As we only conducted one test session for PCS participants, we were not able to determine at what point in time K-D scores worsened. A study by Heitger [26] showed saccadic eye movements among PCS patients to be significantly worse at 140 days PI compared with those with good recovery. Rizzo [32] also examined performance on the K-D test and found significant differences between PCS and healthy controls at a median of 54 weeks PI. It is plausible that differences scores on the K-D test might already arise by 140 days PI and remain elevated past the 1 year mark. To our knowledge, no studies have so far administered the K-D test over multiple test visits in a span of multiple months to over a year PI. Future studies should focus on conducting more frequent visits over time, to better elucidate the pattern of performance on saccadic eye movement tests such as the K-D test among those suffering from chronic concussion.

In terms of symptom scores, we found higher symptom and symptom severity scores among the PCS group compared to both controls and the acute mTBI, suggesting prolongation of symptom burden in this group. A strong possibility is that symptoms related to PCS might not stem from concussion per se but are also influenced by psychological, personality, and psychosocial factors. Individuals react differently to injuries, and it is plausible that those suffering from PCS may develop a shaken sense of identity as concussion recovery takes longer than usual. This may lead to cognitive issues by suppressing attention, mental efficiency, learning and memory, therefore creating symptoms that are unrelated to those caused by the concussion itself [34]. This can in turn lead to frustration and anxiety, leading to avoidance of anxiety provoking situations leading to a buildup of depression that builds over time, resulting in heightened symptom scores [34]. There is also the possibility that symptoms were not specific to PCS, but rather a reaction to the trauma experienced by the head injury [35]. To better elucidate the precise effects of concussion in the development of symptoms in PCS, a control group consisting of patients suffering from non-head injuries would be useful to include in future analyses. In addition, as we also only conducted one study visit, we also cannot say conclusively what the pattern of symptom scores over time in those with PCS are. There has not been sufficient literature conducted so far to provide enough evidence to characterize when elevated symptom scores compared with those with acute mTBI arise and when they subside. Hou [36] did a study in which they found that there was no significant recovery in those with PCS from the 3 to 6-month period PI. McMahon et al. [37] also examined a cohort of individuals with PCS and found that the number of symptoms increased from the 3 to 6 months and from 3 to 12 months PI. Our findings of heightened and persisting symptom scores at a median of 366 days after injury are therefore in line with some of the literature examining PCS, but more studies, examining more frequent visits and over a longer period of time are needed to better characterize symptom scores.

Interestingly, we were not able to show a strong positive correlation between K-D scores and symptom scores. Even more striking is the fact that a statistically significant negative correlation was observed on the first test visit for acute participants. This suggests that the reporting of subjective symptoms does not predict performance on visual based testing scores, and that performance on the K-D test does not predict who experiences greater number of symptoms following injury. It is plausible that the pathways responsible for generating saccades in the brain are independent of those that contribute to behavioral, emotional, and cognitive states, and that both of these cognitive domains contribute to the screening for signs of concussion, albeit separately.

Although we saw total symptom and severity scores improve between the first and second assessments in the acute group, we could not show an improvement in K-D times over the two visits. Previous studies have shown that the K-D test done on the sidelines in sporting contexts could detect abnormalities among affected athletes [1, 2–15]. These abnormalities included factors such as blurred vision or attentional deficits, and the K-D scores reflected this through an increase in scores from pre-injury baseline scores. [1, 2–15]. It could thus be that our acute patients had already recovered rapid eye movements by the time we performed the K-D tests (median 5 days post injury) and so they did not change by the second visit (median 24 days post injury). Neuropsychological tests done after the acute recovery period have found that patients with head injuries have no noticeable test differences from those of matched controls [38, 39]. Silverberg [20] found that the K-D test was not sensitive in differentiating acute mTBI patients and controls at a mean time of 31 hours after injury supporting our thesis that K-D times recover quickly after injury. Our findings therefore do not support the use of the K-D test as a screening test for those with acute mTBI without baseline scores.

Our results also showed the novel and robust finding that K-D times were significantly negatively correlated with the number of words on the Stroop Color and Word Test among all three participant groups. This meant that injured and non-injured participants that completed the K-D test faster got more words correct in 45 seconds on the Stroop Color and Word Test. To date, the K-D has not been studied for its correlation with assessments like the Stroop Color and Word Test. These findings support the idea that the K-D test can also indirectly indicate the level of interference control and selective attention measured by the Stroop Color and Word Test, providing evidence of convergent validity for the K-D test as it also assesses mental abilities such as selective attention. Future studies should consider utilizing these two tests together as the Stroop Color and Word Task can capture additional deficits in attention due to the fact that more strenuous cognitive processes, such as interference control, are often helpful in uncovering signs of concussion in what might be a seemingly non-concussed individual [22–24].

### Limitations and future directions

The interpretation of our study requires a consideration of its limitations. We did not control for the time during the day or the degree of sleep the participant had prior to testing, as they came at their earliest possible convenience. These factors could have produced variations in K-D scores or subjective self-report symptom questionnaires, as previous studies by Fransson [40] and Davies [41] have shown that sleep deprivation can adversely affect eye tracking and attention that is required by the K-D test. Our study design was such that participants could not be assessed immediately following their head injury, as some were admitted into the hospital a couple of hours up until 2 days after their injury. As this was a voluntary study, many patients wanted to be assessed later as they wanted to leave the emergency room of the hospital as soon as their diagnosis was confirmed by a physician. Our mechanisms of injury were varied but when we stratified K-D times according to the mechanism of injury, we did not find any significant differences. Because of the confounding effects of medications, future studies, particularly with PCS patients should control for such medications. Future studies with larger sample sizes may wish to focus on performing an in-depth analysis on the effect of injury mechanism and time since injury, while controlling for a number of confounding factors on K-D times.

Our PCS group, which was not designed to match non-injured controls, was also biased towards females. Our study is in line with previous literature which has reported that those suffering from PCS were more likely to be female, and that there are gender disparities in seeking treatment, which may be reflected in our study [30,34–35]. However, there has not been evidence suggesting that performance on visit based tests is influenced by gender, and that females with PCS score worse than males with PCS. Benedict et al. [21] examined the influence of gender and found it to be associated with increased symptom scores, but not performance on the K-D test. Therefore, although there is bias in gender in those who develop and report PCS, it is unlikely that saccadic performance is correlated with it. Due to our small sample size and difference in number of participants between the PCS and other groups, we are not in the position to draw definitive conclusions about gender and performance on the K-D test. Future studies, with larger sample sizes and with more balanced gender distributions, should explore the option of performing within-group and between-group analyses to better elucidate the effects that gender may play.

Although factors such as motivation can play a role in neuropsychological assessments, we did not consider it necessary to have an effort test to assess motivation, as participants who consented were concerned and eager to participate and contribute to the improvement in treatments for concussion. It is difficult to quantify effort and motivation, and it is an inherent characteristic that varies from individual to individual. To our knowledge, no studies have so far been able to elucidate the effects of effort on performance on visual based assessment tests, nor has a reliable tool been developed for this purpose. Nonetheless, future research should aim to develop an effort test to more accurately control for this potentially confounding factor. Additionally, it is important to note that vision plays only one part in the examination of concussions. The K-D test, as a vision based test, should in appropriate cases, be used as a screening tool and as a compliment to a wider number of assessments in the treatment of concussion. We did not include physical assessments in our study, and future studies should consider using assessments of balance and other tests such as the vestibulo-ocular reflex in the examination of concussions as well in order to more adequately help clinicians screen and diagnose concussion [42]. Larger sample sizes will also allow us to capture acute patients who will develop PCS, to test hypotheses of the sensitivity and specificity of the K-D test in patients with concussion and mTBI over time.

## Conclusions

We found that K-D times inversely correlate highly with the number of correct Stroop words regardless of whether participants had sustained an injury or not suggesting that the K-D test can by-proxy provide insightful information about more complex cerebral functions associated with selective attention, such as response inhibition. We were not able to show any differences in K-D times between acutely injured patients at a median of 5 days post injury and neither did we find times to improve over the subsequent two weeks. In contrast, we showed that PCS patients were slower on the K-D task than controls or acutely injured patients but they were also on multiple medications that might have confounded our results. Our findings do not refute the value of the K-D as a screening test very early after injury. Its use in PCS patients is confounded by factors such as depression frequently seen in patients with PCS. Further large scale studies of the test will better delineate its characteristics acutely and longitudinally.
